# Wood’s Medical and Surgical Monographs

**Published:** 1889-12

**Authors:** 


**Wood's Medical and Surgical Monographs.** Volume IV., No. 2. November, 1889. Published monthly. $10 a year; single copies, $1.

The contents of the current number of this useful series embrace the following titles: On the Surgery of the Knee-Joint, by C. B. Keetley, F. R. C. S.; Aids to Ophthalmic Medicine and Surgery, by Jonathan Hutchison, Jr.; and Bacteriological Technology for Physicians, by Dr. C. J. Salomonsen.

The surgery of the knee-joint, keeping pace with the advances all along the line, has improved to a corresponding degree with that of the other regions of the body. Mr. Keetley is competent authority to speak on this subject, and does so in no uncertain way. He is, moreover, an attractive writer, and forcibly illustrates his points in well-chosen sentences, alluding to the improved surgery of the abdominal cavity and the brain, to illustrate the fact that modern surgery can accomplish marvels, while, in the same breath, he draws the line at miracles.

He quotes from Greig Smith the following, which seems to have an especial force and meaning just now, in view of a recent celebrated case, in which all the modern teachings were grossly violated. Says the distinguished Bristol surgeon:

At once, or within a few hours, we ought to make a definite diagnosis. If we are convinced that it is acute obstruction, then operation should be performed at once; if we are convinced that it is not, another treatment equally definite ought to be pursued. From the beginning, a definite plan of treatment ought to be laid down, and this plan ought to be adhered to. Let it be either drugs or operation, and *never* that *fatal compromise*—operation when drugs fail.

Mr. Jonathan Hutchison, Jr., has, in his Aids to Ophthalmic Medicine and Surgery, which comprises the second part of this number, rendered a valuable service to physicians in placing before them, in a concise manner, certain features of the ophthalmic specialty that should be understood by every practitioner. The chapter on Injuries of the Eye is to be especially commended in this regard.

Bacteriological Technology for Physicians, is the title of Dr. Salomonsen's monograph, which is illustrated with seventy-two figures in the text. The time has arrived when every medical practitioner must know something about bacteriology, and we know of no source whence so much practical information can be derived with so little outlay of time and money as in the study of Dr. Salomonsen's treatise. The relations of microörganism to disease must now be understood, and in order to obtain correct knowledge on this subject, the various kinds of bacteria must be separated and cultivated. Here is the place for the busy practitioner to get the information he must needs have.

